# Physical activity level and lifestyle perception in prebariatric surgery patients

**DOI:** 10.31744/einstein_journal/2019AO4619

**Published:** 2019-06-17

**Authors:** Eduardo Gauze Alexandrino, Danilo Francisco da Silva Marçal, Mateus Dias Antunes, Leonardo Pestillo de Oliveira, Ely Mitie Massuda, Sonia Maria Marques Gomes Bertolini

**Affiliations:** 1 Centro Universitário de Maringá, Maringá, PR, Brasil.; 2 Universidade de São Paulo, São Paulo, SP, Brasil.

**Keywords:** Motor activity, Life style, Obesity, Health promotion, Bariatric surgery, Atividade motora, Estilo de vida, Obesidade, Promoção da saúde, Cirurgia bariátrica

## Abstract

**Objective:**

To determine sociodemographic characteristics, lifestyle perception and physical activity levels in obese prebariatric surgery patients.

**Methods:**

A quantitative, cross-sectional study. The sample comprised 96 male and female morbidly obese prebariatric surgery patients. Questionnaires addressing sociodemographic profile, lifestyle perception and physical activity levels were applied.

**Results:**

Patients were aged 40.3±11.45 years. Inadequate levels of physical activity were reported by 47.8% of patients; most respondents (79.2%) attributed scores defined as inadequate to the physical activity domain of the lifestyle questionnaire. Time spent on physical activity practice per week differed significantly between patients reporting being physically active or physically inactive in adolescence (p=0.046).

**Conclusion:**

Most obese prebariatric surgery patients perceive their lifestyle as inadequate, in spite of eligibility for bariatric surgery. Results also indicate that physical activity practice and nutrition are the domains with greatest impacts on patient lifestyle, and that physical activity practice in adolescence may contribute to adoption of a more active behavior in adulthood, which may represent a vital tool for health promotion in patients undergoing bariatric surgery.

## Resumo

**Objetivo:**

Identificar as características sociodemográficas, a percepção do estilo de vida e o nível de atividade física dos pacientes obesos em pré-cirurgia bariátrica.

**Métodos:**

Pesquisa de abordagem quantitativa, do tipo transversal. A amostra foi composta por 96 pacientes obesos mórbidos pré-cirurgia bariátrica de ambos os sexos. Foram aplicados questionários contendo informações sobre o perfil sociodemográfico, a percepção do estilo de vida e o nível de atividade física.

**Resultados:**

A média de idade dos pacientes foi de 40,3±11,45 anos. O nível inadequado de atividade física avaliado foi verificado em 47,8% dos pacientes, e o domínio atividade física do questionário sobre estilo de vida foi pontuado como inadequado pela maioria dos entrevistados (79,2%). Foram encontradas diferenças estatisticamente significantes entre o tempo de prática de atividade física semanal dos praticantes e não praticantes na adolescência (p=0,046).

**Conclusão:**

A maioria dos pacientes obesos pré-cirurgia bariátrica apresenta percepção de estilo de vida inadequada, apesar de terem sido considerados aptos à cirurgia bariátrica. A prática da atividade física e a nutrição são os domínios que exercem maior impacto no estilo de vida desses pacientes, e a realização de atividades físicas na adolescência pode contribuir para a adoção de um comportamento mais ativo na idade adulta, o que pode representar ferramenta fundamental para promoção da saúde, a ser considerada com os pacientes após a cirurgia bariátrica.

## Introduction

Bariatric surgery aims to support severe obesity treatment.^(1)^ However, the effects of bariatric surgery on this delicate condition are not limited to the surgical procedure. Adoption of a healthier lifestyle before and after surgery is vital for weight maintenance and improved health.^(2)^ Hence the strong association with inadequate performance in several lifestyle domains,^(3)^ characterized by behaviors with positive or negative health impacts.

In practice, treatment for excessive weight is multifactorial and complex. Weight loss is thought to result primarily from lifestyle changes supported by nutritional guidance, use of pharmacological and dietary products, cognitive-behavioral therapy and regular physical activity.^(4)^

Physical inactivity is a major concern in Brazil, given the high prevalence detected in several age groups, particularly among young individuals.^(5)^ Worse still, evidence shows decreasing physical activity levels with age and weight regain in bariatric patients within 24 months of surgery.^(6)^

Individuals who were physically active in childhood and adolescence are more likely to practice physical activities in adult life.^(7)^ However, the paucity of studies analyzing this variable in morbidly obese individuals prior to bariatric surgery preclude the development of effective public policies for this population.

Individuals who were active in adolescence tend to remain more active over the course of remaining life cycles and are more likely to adhere to regular physical activity practice after bariatric surgery. Bariatric surgery via the Brazilian Public Health System (SUS - *Sistema Único de Saúde*) is limited to eligible patients. Patient eligibility is based on multidisciplinary assessment, psychological soundness certification and likelihood of adopting an adequate lifestyle before and after surgery.^(8)^ Weight regain in patients submitted to bariatric surgery emphasizes the existing gap between theory and practice, a major reason for conducting this study.

Past behaviors, physical and social characteristics of prebariatric surgery patients must be investigated for proper implementation of effective long-term interventions. This study aims to contribute to data related to the population of individuals deemed eligible for bariatric surgery in Brazil.

## Objective

To determine lifestyle perception and physical activity levels in obese patients prior to bariatric surgery.

## Methods

A quantitative cross-sectional study approved by the Research Ethics Committee of *Centro Universitário de Maringá*, opinion no. 1.359.832/2015, CAAE: 51742215.9.0000.5539, in compliance with resolution 466/12 of *Conselho Nacional de Saúde* [National Health Council].

The target population comprised morbidly obese male and female individuals seeking bariatric surgery via SUS and linked to the *Associação dos Obesos e Operados Bariátricos de Paranavaí e Região*. Individuals were duly instructed and screened at that entity. Eligible individuals were then added to the SUS bariatric surgery waiting list and operated according to surgical bed availability. Prebariatric surgery patients were followed up by a multidisciplinary team at two hospitals, *Santa Casa de Paranavaí* and *Hospital Noroeste do Paraná*.

Male and female adult patients aged over 18 years eligible for bariatric surgery and about to complete the final multidisciplinary intervention steps preceding bariatric surgery were included. Patients with a history of recent (last 2 years) pregnancy or using corticosteroids were excluded.

Data were collected at *Hospital Noroeste do Paraná* in March and April 2016.

Patient socioeconomic profile was determined through a questionnaire (*Questionário de Classificação Econômica; Associação Brasileira de Empresas de Pesquisa http://www.abep.org/criterio-brasil*).

Lifestyle, physical activity-related behaviors and weekly preoccupation were investigated using the Fantastic Instrument questionnaire proposed by the Canadian Physical Activity Fitness and Lifestyle Appraisal (CPAFLA) in 1998, and validated for the Brazilian population by Añez et al.^(9)^

Physical activity level assessment was based on the short version of the International Physical Activity Questionnaire (IPAQ). Physical activity levels were rated sedentary, irregularly active, active or very active, and grouped as follows: “adequate” – active and very active individuals; “inadequate” – insufficiently active and sedentary individuals. Physical activity experiences in childhood and adolescence were investigated using a reminder containing two open questions adapted from Fernandes et al.,^(10)^ and seven objective questions.

In this study, active individuals in childhood (7 to 10 years) and adolescence (11 to 17 years) were those providing positive answers to two questions: “From 7 to 10 years of age, out of school, were you engaged in supervised sport activities of any kind for at least one year uninterruptedly?” and “From 11 to 17 years of age, out of school, were you engaged in supervised sport activities of any kind for at least one year uninterruptedly?”.

Data normality was investigated using the Kolmogorov-Smirnov test. Comparative analysis of anthropometric differences, lifestyle scores and variables associated with current and past physical activity practice between sexes and remaining sociodemographic variables was performed using the Mann-Whitney U or the Student’s *t* test (non-parametric and parametric data, respectively). Associations between categories were investigated using the χ^2^ test. The level of significance was set at 5% (p<0.05).

## Results

Data from 96 patients were collected. Sociodemographic characteristics of the sample studied are presented in table 1.

**Table 1:** Sociodemographic characteristics of pre-operative bariatric patients

Variables	
Sex	
Female	84
Male	12
Age	40.3 (11.5)
Women	38.3 (9.8)
Men	42.3 (13.1)
Married	45.8
*De facto* relationship	88.5
Occupationally active	67.7
Earnings between 2 and 3 minimum wages	52.1
Secondary education or incomplete higher education	38.5

Results expressed as n, mean (standard deviation) or %.

Individuals rated adequate regarding current physical activity levels had lower body mass index compared to those rated inadequate (Table 2).

**Table 2:** Comparison of age and body mass index (BMI, kg/m2) with regard to physical activity practice in adolescence, presence of chronic diseases, obesity and physical activity level in morbidly obese patients

Variables		Yes (n=30)	No (n=66)	p value
Physical activity in adolescence	Age, years	34.5±11	40.7±9.4	0.001*
	Age of weight gain	15.7±9.9	18.3±9	0.044*
	BMI	43.4±7.4	44.1±5.3	0.079
		Yes (n=62)	No (n=34)	
Chronic disease	Age, years	40.1±11.1	36.6±8.4	0.122
	Age of weight gain	17.8±10	16.9±8.1	0.395
	BMI	44.84±6.56	42.15±4.53	0.023*
		Grade II (n=30)	Grade III (n=66)	
Obesity	Age, years	36±8.7	40±10.8	0.030*
	Age of weight gain	18.1±9.2	17.2±9.4	0.302
	BMI	37.9±1.5	46.6±5.3	0.001*
		Adequate (n=47)	Inadequate (n=49)	
Current physical activity level	Age, years	36.3±8.3	41.2±11.5	0.012*
	Age of weight gain	16.8±8	18.2±10.4	0.408
	BMI	43±5	44.8±6.9	0.096

Results expressed as mean±standard deviation. Mann-Whitney U test. * Significant values p≤0.05.

As shown in table 3, morbidly obese individuals of high socioeconomic class with no musculoskeletal disorders and reporting regular practiced of physical activities and exercises achieved significantly higher lifestyle scores.

**Table 3:** Comparison of mean lifestyle scores with regard to sociodemographic variables, current physical activity levels and musculoskeletal disorders in prebariatric surgery patients

Variables	Fantastic Instrument Score	p value
Sex		0.295
Female, n=84	66.1±9.1	
Male, n=12	61.8±13.4	
BMI, kg/m^2^		0.281
Grade II, n=30	66.6±6.1	
Grade III, n=66	65±11	
Chronic disease		0.066
Yes, n=62	64.2±8.9	
No, n=34	68±10.8	
Occupational status		0.311
Active, n=65	66.3±10.4	
Inactive, n=31	64.1±8.2	
Income		0.172
≤1 MW, n=34	64.2±9.8	
>1 MW, n=62	66.3±9.7	
Socioeconomic class		0.027*
High, n=40	68.2±9.4	
Low, n=56	63.7±9.6	
Musculoskeletal disorders		0.038*
Yes, n=35	62.5±8.7	
No, n=57	67.5±10.1	
Regular physical activity practice		0.032*
Yes, n=35	68.4±8.9	
No, n=61	64±9.9	
Current running practice		0.024*
Yes, n=34	68.7±8.8	
No, n=62	63.3±9.9	
Sports practice/leisure		0.009*
Yes, n=10	73.1±7.9	
No, n=86	64.7±9.6	
Regular physical exercise practice		0.048*
Yes, n=6	73.2±9	
No, n=90	65.1±9.6	
Current general fitness training		0.048*
Yes, n=6	73.2±9	
No, n=90	65.1±9.6	
Current weight lifting exercises		0.048*
Yes, n=2	79±1.4	
No, n=94	65.3±9.6	

Portuguese version: *Estilo de Vida Fantástico*.

Results expressed as mean±standard deviation. Weight lifting exercises and general fitness training under professional guidance were grouped as physical exercise; regular running and leisure sports practice without professional guidance were grouped as physical factitivo; Student‘s *t* test.

*Significant values p≤0.05. BMI: body mass index; MW: Minimum wage.

In obese individuals whose lifestyle was rated “adequate”, associations between lifestyle and remaining variables were limited to musculoskeletal disorders (p=0.003) and physical activity practice (p=0.012).

Analysis of current physical activity practice according to sex revealed that time spent sitting over the weekend was longer among male individuals (p=0.042).

As regards physical activity levels according to IPAQ, 52.20% of patients practiced adequate levels of physical activity. Comparative analysis of regular physical activity practice with regard to physical activity level, physical activity practice in adolescence, presence of chronic diseases and occupational status of prebariatric surgery patients living in Paranavaí (PR) yielded relevant results.

Physical activity levels differed significantly between patients with levels rated adequate and inadequate: patients with adequate physical activity levels were defined as those who practiced more physical activities during the week (p=0.001), exercised more days per week (p=0.001) and spent less time sitting during the week (p=0.007). However, time spent sitting during the day did not differ significantly (p=0.212), indicating that physical activity should be assessed in several consecutive days rather than a single day.

As regards physical activity practice in adolescence, significant differences were limited to weekly physical activity practice (more common among patients practicing physical activities in adolescence; p=0.046). In contrast, physical activity practice during the week was significantly less frequent in patients suffering from chronic diseases compared to those who did not suffer from chronic diseases (p=0.007). Finally, occupationally active patients were also more physically active during the week, reporting 2 days more of physical activity practice on average compared to occupationally inactive patients (p=0.018).

Lifestyle was rated adequate by 30.2% of patients only. Associations between regular physical activity practice and lifestyle is showed in table 4.

**Table 4:** Lifestyle and regular physical activity levels as perceived by morbidly obese prebariatric surgery patients

Variables	Lifestyle		p value
Physical activity level	Adequate	Inadequate	
Adequate	20 (20.8)	27 (28.1)	0.010*
Inadequate	9 (9.4)	40 (41.7)	
Physical activity practice			
Yes	16 (16.7)	19 (19.8)	0.012*
No	13 (13.5)	48 (50.0)	

Results expressed as n (%). * Significant values p≤0.05. χ^2^ test.

Physical activity levels and lifestyle perception were positively associated with physical activity practice. The lifestyle of patients influenced and was influenced by physical activity practice, supporting the importance of physical activity in people’s life.

## Discussion

This study revealed inadequate levels of physical activity in 51.4% of patients in the sample. According to the physical activity domain of the lifestyle questionnaire, most interviewees (69.79%) fell in the inadequate category. These are alarming and concerning data, as they reflect screened patients deemed eligible for bariatric surgery following multidisciplinary assessment, psychologically sound and capable of adopting an adequate lifestyle before and after surgery.

This study also showed that obese individuals who were physically active in adolescence dedicated more time to physical activities per week. This finding underscores previous research reporting that individuals with a history of regular physical activity practice in adolescence scored higher on IPAQ variables.^(6)^ Physical activity practice in adolescence seems therefore to contribute to retention of this behavior in adult life in spite of morbid obesity; physical activity practice is a key factor in lifestyle changes aimed to maintain weight loss and prevent weight regain after bariatric surgery.

Excess weight gain occurred primarily in adolescence in patients in this sample. Growing evidence indicates that obesity resulting from inadequate lifestyle in this age group tends to persist in adult life.^(4,5)^

Musculoskeletal disorders have a negative impact on lifestyle perception among obese individuals.^(11,12)^ In this study, patients reporting musculoskeletal disorders achieved significantly lower lifestyle scores.

Inadequate eating behaviors observed in this study support results reported elsewhere,^(13)^ emphasize the inability of patients to adhere to advice given by multidisciplinary teams, and reveal that risky eating behaviors and sedentary lifestyle stem from childhood. Hence the consensus that bariatric surgery requires strong adherence to post-surgical dietary and behavioral changes on the part of patients to ensure treatment efficacy.^(6)^

Limitations of this study include small numbers of male individuals in the sample, as in other studies,^(1)^and cross-sectional study design precluding investigation of causal relations between variables. However, in spite of aforementioned limitations, this study provides one of the few reports on physical activity levels in obese individuals.

Findings of this study regarding lifestyle perception and physical activity levels in obese prebariatric surgery patients suggest that the objectives were successfully met. Patient characteristics constituted an interesting factor for development of strategies aimed to improve surgical outcomes. Most patients perceived their lifestyle as inadequate; still, they were all deemed eligible for surgery.

Physical activity practice may be singled out as the one variable with greatest impact on patient lifestyle and may represent a vital tool for health promotion in prebariatric surgery patients.

## Conclusion

Most Obese Prebariatric Surgery Patients Had Inadequate Lifestyle Perception In Spite Of Eligibility For Bariatric Surgery And Adequate Levels Of Physical Activity.
